# A potential new mechanism for pregnancy loss: considering the role of LINE-1 retrotransposons in early spontaneous miscarriage

**DOI:** 10.1186/s12958-020-0564-x

**Published:** 2020-01-21

**Authors:** Chao Lou, John L. Goodier, Rong Qiang

**Affiliations:** 1 Department of Genetics, Northwest Women’s and Children’s Hospital, 1616 Yanxiang Road, Xi’an, Shaanxi Province People’s Republic of China; 2 McKusick-Nathans Deartment of Genetic Medicine, Johns Hopkins University School of Medicine, Baltimore, MD USA

**Keywords:** Spontaneous miscarriage, Retrotransposon, LINE-1, *De novo* insertion, Human embryogenesis, Mutation

## Abstract

LINE1 retrotransposons are mobile DNA elements that copy and paste themselves into new sites in the genome. To ensure their evolutionary success, heritable new LINE-1 insertions accumulate in cells that can transmit genetic information to the next generation (i.e., germ cells and embryonic stem cells). It is our hypothesis that LINE1 retrotransposons, insertional mutagens that affect expression of genes, may be causal agents of early miscarriage in humans. The cell has evolved various defenses restricting retrotransposition-caused mutation, but these are occasionally relaxed in certain somatic cell types, including those of the early embryo. We predict that reduced suppression of L1s in germ cells or early-stage embryos may lead to excessive genome mutation by retrotransposon insertion, or to the induction of an inflammatory response or apoptosis due to increased expression of L1-derived nucleic acids and proteins, and so disrupt gene function important for embryogenesis. If correct, a novel threat to normal human development is revealed, and reverse transcriptase therapy could be one future strategy for controlling this cause of embryonic damage in patients with recurrent miscarriages.

## Background

Spontaneous abortion or miscarriage is defined as natural death of an embryo or fetus before the twentieth week of pregnancy (the term stillbirth is used after 20 weeks). Most miscarriages occur during the first 7 weeks when the embryonic trophoblast invades the endometrium in a process analogous to tumor invasion and metastasis. Among clinically confirmed pregnancies, the incidence of spontaneous miscarriage is about 15 percent. However, it is estimated that about 50 to 75 percent of total pregnancies are miscarried. Among these, most of the aborted embryos cease development soon after implantation, appearing as menorrhagia or delayed menstruation, and escape notice (reviewed in [1, 2]).

Numerous causes of spontaneous abortion have been identified, including maternal reproductive tract abnormalities, endocrine and immunological dysfunction, sperm issues, reproductive tract infections, cervical insufficiency, thrombophila, and chromosome abnormalities, among others [1, 3]. Abnormal chromosome karyotype is seen in about 50% of spontaneous abortion patients, with triploidy most common, followed by autosomal unbalanced translocation, and polyploidy, X monomer, autosomal monomer, chromosome balanced translocation, deletion, chimerism, inversion, overlap, and so on [4, 5]. During embryonic development a single lethal gene mutation may also lead to death of the embryo [6]. Furthermore, evidence suggests that epigenetic anomalies may lie behind some cases of early pregnancy loss [7]. Recently, the key role that the placenta exerts on embryo development has been uncovered, adding another layer of complexity to the miscarriage phenomenon [8]. However, in the case of recurrent pregnancy loss, defined as at least three consecutive miscarriages prior to 24 weeks gestation [9], cause can be identified in only about 50 percent of cases [10]. In general, the genetic causes of miscarriage are poorly understood: much more study is required.

Here we propose the hypothesis that Long Interspersed Element-1 (LINE-1 or L1) retrotransposon activity may be a previously unrecognized causal factor for some cases of spontaneous miscarriage in humans. We suggest that during the development of gametes or human embryos, increased LINE-1 genomic insertions may disrupt one or more genes critical for early human embryonic development leading to miscarriage. Retrotransposon insertions may also mediate chromosomal rearrangements and alter the local epigenetic environment, among other effects. Furthermore, as discussed below, there is increasing evidence that, apart from insertion mutation, elevated L1 expression, especially of its reverse transcriptase (RT) and endonuclease activities, may initiate DNA damage or an immune response [11, 12]. Such phenomena could lead to embryo damage.

**Fig. 1:**
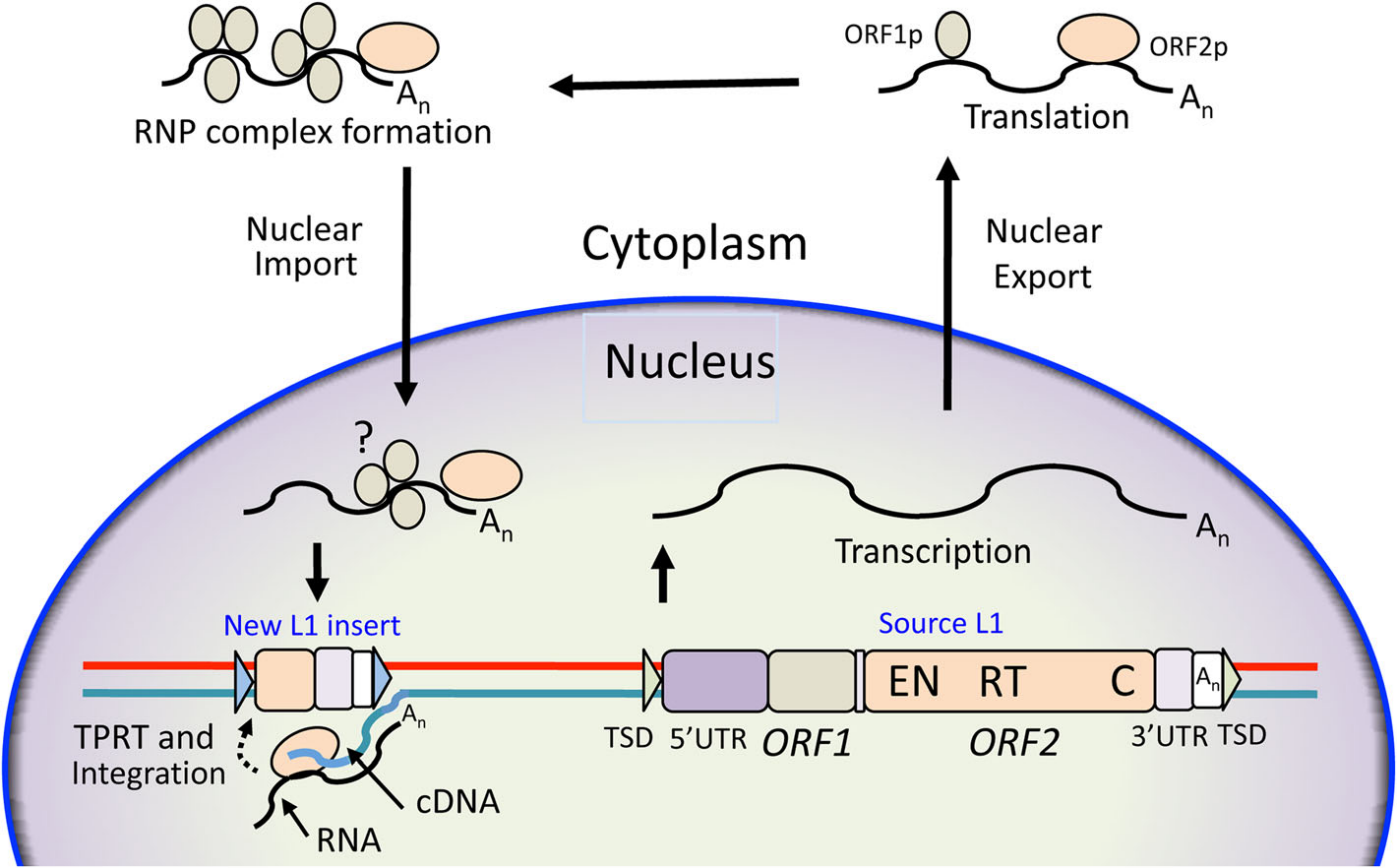
The biology of a LINE-1 retrotransposon. The structure of a human L1 is shown. TSD: target site duplication; UTR: untranslated region; EN: endonuclease; RT: reverse transcriptase; C: carboxy-terminal segment; A_n_: polyadenylation signal and tail. The LINE-1 replication cycle involves transcription and export of its RNA to the cytoplasm, which is translated and assembled in a ribonucleoprotein particle (RNP) together with L1 ORF1p and ORF2p. There is a strong *cis*-preference for L1 ORF1 and ORF2 proteins to bind their own encoding RNA in a retrotransposition-competent RNP. ORF1p binds L1 RNA as a trimer, however, it is unclear if it remains bound at the time of import of the RNP into the nucleus (denoted by ?) ) [20–22]. Reverse transcription of LINE-1 RNA to generate complementary (cDNA) occurs at the site of chromosomal insertion by TPRT [23]. L1s frequently become 5′-truncated when inserted in the genome

It has been estimated that over two-thirds of the human genome is repetitive DNA, most of this transposable elements (TEs) [13]. There are two main classes of TEs in genomes. Class II elements, the DNA transposons, replicate by a “cut and paste” mechanism, although no active transposons exist in humans. Class I elements, the retrotransposons, move by a “copy and paste” mechanism involving reverse transcription of an RNA intermediate and insertion of its cDNA copy at a new site in the genome. There are two major subgroups of Class I elements: long terminal repeat (LTR) and non-LTR retrotransposons. LTR retrotransposons include endogenous retroviruses (ERVs), relics of past rounds of germline infection by viruses that lost their ability to reinfect new cells. Human (H)ERVs compose 8% of our genome, although no remaining retrotransposition-competent HERVs have been identified. Nevertheless, genetic evidence suggests recent HERV activity in humans, and some HERV-K(HML-2) copies are polymorphic in the human population [14–16]. In humans the only autonomously active TE is LINE-1 (L1), a non-LTR retrotransposon with approximately half a million copies occupying about 17% of our genome [17]. L1s have also been responsible for the insertion *in trans* of over ten thousand processed pseudogenes and a million non-autonomous Short Interspersed Elements (SINEs), including Alu and SINE-VNTR-Alu (SVA) elements [18, 19]. A full-length active six kilobase bicistronic human L1 contains two non-overlapping open reading frames (ORFs) that encode the RNA-binding ORF1 protein (ORF1p) and the longer ORF2p, which functions both as a reverse transcriptase and DNA endonuclease (Fig. 1). Retrotransposition of a non-LTR retrotransposon is fundamentally different from that of an ERV, whose replication cycle involves reverse transcription of its genome in the cytoplasm. L1-encoded endonuclease nicks the bottom strand of target chromosomal DNA exposing a 3′-hydoxyl group that primes reverse transcription of the L1 RNA and synthesis of cDNA bound at the site of insertion, a process known as target primed reverse transcription (TPRT) [23].

Most L1s are 5′ truncated and otherwise rearranged or mutated, and hence are incapable of retrotransposition. However, it is considered that about 100 LINE-1 sequences are full-length with intact ORFs and potentially active, although fewer than ten are considered to be “hot” and these consistently account for the bulk of new retrotransposition in humans [24–27]. Up to 5% of newborn children have a new retrotransposon insertion, and to date there have been 125 known human disease-causing germline non-LTR retrotransposon insertions [28–32]. The genomic revolution, including high-throughput (HT) sequencing analyses, has allowed estimates of the rates of L1 retrotransposition in mammals; indeed, recent studies indicate that a new L1 insertion may occur in 1 in 62 human births (1 in 40 births in the case of Alus), and 1 in 8 births in mice [33, 34]. The cell has evolved a battery of defenses to protect against unfettered retrotransposition (reviewed in [35, 36]). However, in some somatic cell types or under certain cellular conditions the defenses are lowered and retrotransposition increases.

### Retrotransposon activity and its control in early embryonic development

In addition to the massive germ line expansion of L1s that occurred during mammalian evolution, recent investigations have documented ongoing retrotransposition in some somatic cell types, including neural progenitor cells, some tumors, stem cells, and notably early embryos (reviewed in [37–49]). Transgenic mouse and human studies demonstrated that somatic retrotransposition occurs in early-stage embryos causing somatic mosaicism [33, 50–53]. Cultured human embryonic stem cells (ESCs) and induced pluripotent stem cells (iPSCs) express endogenous L1 RNA and proteins and support both retrotransposition of transfected reporter constructs [54–60] and modest levels of endogenous retrotransposition [61–63]. Recently, Muñoz-Lopez et al. [63] showed expression of non-LTR retrotransposons in the inner cell mass (ICM) and trophectoderm cells of pre-implantation human embryos and, using HT sequencing, *de novo* endogenous LINE-1 insertions within cells of the ICM as well as insertions restricted to the placenta. Thus, the cellular environment of early embryonic cells supports active retrotransposition. Of course, activity during early embryogenesis is beneficial for the evolutionary success of the L1, as new insertions have a high chance of being transmitted to the next generation.

Various cellular mechanisms restrict retrotransposition in the germline and embryos. For example, small interfering RNA (siRNA)-mediated gene silencing is an ancient strategy for controlling activity of TEs. RNA interference acts at the post-transcriptional level by causing RNA degradation and loss of translation, or at the transcriptional level by causing epigenetic modifications, including *de novo* methylation of TE sequences. piRNAs are small RNAs found in testes as well as human fetal ovaries that specifically silence TEs in the germline ( [64]; reviewed in [65–69]). A large percentage of mouse prepachytene piRNAs derives from retrotransposon sequences [70, 71], and the importance of piRNA pathway proteins in repressing retrotransposon expression in prenatal gonad development and spermatogenesis has repeatedly been demonstrated in mutant mouse lines defective for piRNA pathway proteins (reviewed in [36]).

It has been proposed that DNA methylation of CpGs evolved primarily as a host defense mechanism against TEs [72, 73]. Indeed, the L1 promoter is a prototypical CpG island and L1 promoter methylation is inversely correlated with L1 expression [74, 75]. In early mouse embryogenesis, repression of retrotransposons is maintained by histone and DNA methylation. However, successive waves of demethylation occur in the developing embryo and open windows for increased retrotransposon activity [76–78]. The first wave occurs shortly after fertilization until the morula stage. Around E8.5, demethylation occurs again in post-implantation primordial germ cells (PGCs) and continues to around E13 when PGCs have colonized the genital ridges (summarized in [79–82]).

The promoters of young active L1 elements are hypomethylated in hESCs compared to differentiated cells, which accounts in part for their higher levels of expression [83, 84]. In the case of embryonic tissues, human L1 methylation status has mostly been studied for the placenta, and both hypermethylation and hypomethylation have been reported. According to one study, LINE-1 methylation is significantly decreased in third trimester compared with first trimester placentas, a trend not paralleled by change in global methylation [85]. Perrin et al. [86] found that, compared with unaffected individuals, LINE-1 hypermethylation during development and differentiation of the placenta is two-fold higher in human hydatidiform mole patients, a condition involving abnormal placental growth and spontaneous abortion; methylation of other repeats and global methylation did not differ. Vasil’ev et al. [87] observed increased LINE-1 methylation in placental tissues of spontaneous abortions having mosaic aneuploidy but not in miscarriages with complete aneuploidy or in induced abortions. On the other hand, in extraembryonic tissues of spontaneous abortions with normal karyotype, LINE-1s were excessively hypomethylated. LINE-1 hypomethylation can result in enhanced L1 activation and consequent mutational insertions. Consistent with this hypothesis, Sanchez-Luque et al. [84] recently uncovered the critical role for DNA methylation in controlling activity of “hot” L1s in humans.

### Many genes are involved in early embyogenesis

In principle, a new L1 insertion into a lethal gene could initiate a cascade leading to fetal death, although our diploid nature limits such consequences. Many signaling pathways and genes are involved in the process of miscarriage and single gene mutations may cause spontaneous abortion [6]. Based on a study of 489 single gene knockout mouse models, White et al. [88] found 29 percent of the genes to be lethal and 13 percent sublethal. *KIF7* (kinesin family member gene 7) was the first human gene associated with fetal lethality when it was found to cause hydrolethalus and acrocallosal syndromes [89], and since then many other candidate genes have been identified. A review of 50 human studies identified a range of possible causative gene and copy number variations (CNVs) for miscarriage, including *CHRNA1* (cholinergic receptor, nicotinic, alpha polypeptide 1)*, DYNC2H1 (*dynein, cytoplasmic 2, heavy chain 1), and *RYR1 (*ryanodine receptor 1)*,* which were reported by multiple studies [6]. Several whole exome sequence analyses of euploid miscarriages have been conducted, including a study of 30 fetuses in which mutations in *FGFR3* (fibroblast growth factor receptor 3), *COL2A1* (collagen, type II, alpha 1), and *OFD1* (oral-facial-digital syndrome 1) genes, in addition to structural variants, accounted for 10 percent of the cohort [90]. Fang et al. [91] found that expression of VEGF (vascular endothelial growth factor), part of the angiogenesis signaling pathway, was significantly decreased in missed abortion tissue and correlated with increased levels of VEGFR1 (Vascular Endothelial Growth Factor Receptor 1) and Notch-1. Adache et al. [92] reviewed the key role of the cyclooxygenase (COX)-1 and -2 signaling pathways for repeated failure of embryo implantation. Affected genes found in other studies include KIF14 (kinesin family member 14) [93], IFT122 (intraflagellar transport 122) [94], PLCD4 (phospholipase C delta 4), and OSBPL5 (protein-like 5) [95]. In the case of recurrent miscarriage, cytokine gene polymorphisms, novel HLA alelles, and mutations in inflammatory factors and synaptonemal complex protein 3 (SYCP3) have been implicated. SYCP3 encodes an essential structural component of the synaptonemal complex and its mutation may result in chromosome abnormalities [96–99]. Thus, it is increasingly evident that mutation of any of many cellular pathway genes can initiate miscarriage.

Studies have demonstrated that healthy humans carry many mutated gene alleles [100]: elevated L1 retrotransposition during early embryogenesis could contribute to this mutation burden. It is possible that during early development epigenetic change or loss of a retrotransposon inhibiting factor could trigger derepression of active retrotransposons increasing the likelihood of an L1 inserting into a lethal gene. Recent studies have revealed the complexity of cellular factors and pathways regulating the activity of human retrotransposons. To date about 80 factors have been identified that limit expression or insertion of retrotransposons in cell culture or mouse models ([101]; reviewed in [36]). For example, knockout of DNA Methyltransferase 3 Like (DNMT3L) protein in mouse germ cells was accompanied by epigenetic change, reactivation of retrotransposons, and meiotic collapse [77]. Loss of TEX19.1 in mice leads to placental growth retardation, increased embryonic lethality, and derepressed retrotransposon expression in placenta and hypomethylated trophectoderm-derived cells, and its loss in mouse pluripotent embryonic stem cells increases retrotransposition of engineered L1 constructs [60, 102]. To cite another example, using a digital droplet PCR detection strategy, a startling 70-fold increase in retrotransposition of an L1 reporter transgene in a mouse deficient for MOV10L1, a piRNA pathway protein, was claimed by Newkirk et al. [103].

The impacts of retrotransposons on gene integrity extend beyond simple mutation by insertion: these have been the subjects of many reviews [18, 32, 104–107]. Ongoing retrotransposition events salt genomes with novel splice sites, polyadenylation signals, promoters, and transcription factor binding sites that can alter gene expression. Recombination between retrotransposons causes deletions, duplications, or rearrangements of gene sequence, and this is especially true for Alus [108]. L1-mediated retrotransposition insertion can also cause deletions up to a megabase at their sites of insertion [18, 105, 109–112]: one example is the deletion of an entire HLA-A gene caused by an SVA insertion that resulted in leukemia [113]. Retrotransposons are also associated with segmental duplications [114]; significantly, CNVs have also been linked with human miscarriage [115, 116]. Even more dramatic non-LTR retrotransposon-mediated genomic rearrangements may occur. L1 endonuclease activity and SVA retrotransposition leading to multiple DNA breaks was proposed as causal for one case of human germline chromothripsis [117], a phenomenon involving numerous chromosomal rearrangements in a single event, and one that has also been linked with severe congenital defects [118]. In summary, the mutagenic potential of active human retrotransposons can be significant.

### A possible role for misregulation of retrotransposon expression in embryonic failure

Apart from insertion mutation, various studies have proposed physiological roles for retrotransposon expression, and these roles may turn pathological when expression is misregulated. Significant research has centered on the cellular effects of reverse transcriptase with implications for the developing embryo.

Functional RT activity has been reported in mature spermatozoa and pre-implantation embryos of mice [119–121]. Treatment of early stage mouse embryos with either antisense L1 oligonucleotides, an antibody to RT, or the RT inhibitor nevirapine reportedly arrested preimplantation development at the 2- to 4-cell stage, perhaps by altering levels of cellular cDNA synthesized by RT [120, 122]. (However, It should be noted, non-nucleoside reverse transciptase inhibitors like nevirapine, while they inhibit ERVs, were subsequently shown to not inhibit L1 cell culture retrotransposition [123–125]).

More recently, using antisense oligonucleotides to deplete L1 transcripts, Percharde et al. [126, 127] presented evidence that LINE1 expression plays a role in mouse embryonic exit from the 2-cell stage by recruiting nucleolin and Kap1 to repress the master transcriptional regulator Dux and activate rRNA synthesis. Furthermore, Jachowicz et al. [128] reported that LINE-1 activation after fertilization regulates global chromatin accessibility, and that artificial prolongation of L1 transcription in mouse embryos interferes with their development. Thus, both teams obtained comparable results after altering LINE-1 expression in mouse embryos, suggesting that proper functioning of a potential mutagen paradoxically also plays a role in embryonic development.

Elevated expression of an L1 transgene in mice null for Maelstrom, a piRNA pathway gene, was associated with increased meiotic prophase I defects, DNA damage, and fetal oocyte attrition [129, 130]. Oocyte attrition is a mysterious process involving loss of about two-thirds of human meiotic prophase oocytes [131]. The fact that treating mice with a nucleoside analog blocked oocyte attrition suggests roles for retrotransposon RT and perhaps endonuclease activities. As a normal part of TPRT, L1 ORF2 endonuclease generates dsDNA breaks that recruit repair proteins to the site of element insertion. However, transient transfection of an L1 in cell culture has been reported to induce DNA breaks manyfold in excess of what would be expected for TPRT-mediated insertions alone, and DNA damage caused by overexpression of ORF2p can induce genotoxic stress and cell death [132–134].

Recent evidence suggests that cellular conditions that stimulate increased expression of L1s, and therefore ORF2 protein and its RT, may generate ectopic retrotransposon cDNAs not engaged in TPRT at the site of genome integration. For example, aged cells and mice accumulate cytoplasmic L1-derived cDNAs, triggering an interferon response as a result of misidentification of these self-derived nucleic acids as non-self, while treatment with reverse transcriptase inhibitors reduces inflammation and increases viability and lifespan [135, 136]. Thomas et al. [137] also reported an interferon response and toxicity associated with accumulation of extrachomosomal L1-related single-stranded DNAs in neurons derived from hESCs lacking TREX1, a DNA exonuclease mutated in patients with Aicardi-Goutières syndrome (AGS), a rare childhood Type I interferonopathy involving loss of brain white matter [138].

While some studies have suggested that interferons play crucial roles in mammalian pregnancy, abnormal inflammatory reactions have also been associated with early pregnancy loss (reviewed in [139, 140]). Higher levels of Th1-type or pro-inflammatory cytokines, including IFNγ, were found in women with recurrent miscarriage when compared with women with normal pregnancies [141, 142]. Whether increased expression of retrotransposon-encoded RT can induce an interferon response in the developing embryo remains to be tested.

### Testing the hypothesis

Recent years have seen the development of various HT sequencing strategies that could be applied to detection of *de novo* non-LTR retrotransposon insertions in genomic DNA of miscarriage samples. These include hybridization-based enrichment methods (including RC-seq [143]), selective PCR amplification (including ATLAS-Seq, L1-Seq, TIP-seq, and other methods [144–150]), and algorithms to analyze whole genome sequence (including The Transposable Element Analyzer (Tea), TEBreak, The Mobile Element Locator Tool (MELT), and others (https://github.com/adamewing/tebreak; [31, 151–156]). Candidate insertions are compared with insertions detected in the reference human genome, databases of non-reference polymorphic retrotansposons (such as dbRIP and euL1db [157, 158]), and parental blood DNA sequence to ascertain that the insertions occurred during development of the embryo or within the parental germline. One should further validate the insertions by site-specific PCR and Sanger sequencing of the amplicons to confirm the exact location of the 3′ and 5′ junctions. The best candidate tissues for initial testing for retrotransposon-caused defects may be recurrent miscarriages, which affect 1 to 2 percent of couples and for which cause can be identified in only half of cases [10, 159–161]. If available fetal tissue amounts are limited, primary cell lines may be derived and expanded in culture. Alternatively, and despite significant challenges [162], single cell genomics may be used to identify new L1 insertions in miscarriage samples. Of course, studies to assess retrotransposon insertions in early human embryonic development may be frustrated by access to tissues, so alternatively transgenic mouse models for L1 retrotransposition can be useful [51, 53, 163–165].

L1 RNA expression in miscarriage-related samples may be assessed by RT-qPCR, Northern blotting, RNA FISH, and RNA-Seq methods. A number of papers discuss the analysis algorithms, special protocols, difficulties, and caveats to be considered when analyzing expression of high-copy number retrotransposon loci with highly similar sequences [42, 83, 165–170]. Changes in L1 protein levels or patterns of subcellular distribution may be assayed using immunohistochemistry and Western blotting. Many labs have developed effective L1 α-ORF1p antibodies; we recommend the 4H1 α-ORF1p antibody available from MilliporeSigma [171]. Endogenous L1 ORF2p is expressed at very low levels and few effective antibodies have been reported [172–174].

If increased retroelement mRNA and proteins are detected in miscarriage samples, one would predict an increase in RT activity with possible consequences for the cell, as noted above. Various assays have been established to detect RT activity in cells, whether deriving from L1 ORF2p or HERV pol genes [175–177]. Using RT-qPCR to assay changes in expression of interferon-stimulated genes may also reveal autoinflammatory effects of retrotransposon misregulation, as described above for AGS and some other autoimmune conditions [137, 178–180].

If this hypothesis is supported, that retrotransposon activity significantly contributes to fetal damage in some patients, ameliorative options are conceivable. Administration of low doses of RT inhibitor to such patients could reduce the incidence of future retrotransposition and miscarriage. In cell culture experiments, L1 retrotransposition is strongly inhibited by nucleoside reverse transcriptase inhibitors (NRTIs) and recent studies have identified NRTIs that limit L1s and/or HERVs, including drugs widely used against HIV-1 infection [123–125]. Of interest, pilot clinical trials using NRTI inhibitors to reduce retrotransposon activity have begun for amyotrophic lateral sclerosis (ClinicalTrials.gov Identifiers NCT02437110, NCT02868580, [181]) and AGS (NCT02363452, NCT03304717). One of the AGS trials, now completed, reported reduction in interferon-stimulated gene expression in treated patients [182].

In summary, we propose that increased LINE-1 activity may be one cause of spontaneous miscarriage. This concept is reasonable according to the points outlined above, and especially considering the reported involvement L1 RNAs in proper preimplantation embryo development [126, 128] and the increased activity of L1s in early human embryos [63]. Deleterious cell effects of elevated retrotransposon activity may involve L1-mediated gene disruption by insertion mutation or the initiation of inflammatory or DNA damage responses. However, as for oocyte attrition in mice [129], it is possible that human embryos typically clear damaged embryonic cells by apoptosis and related mechanisms. If active L1s are indeed involved in miscarriage, it would increase understanding of spontaneous miscarriage mechanisms and have clinical significance for pregnant women. LINE-1 insertions may become a new reason given to miscarriage patients, and such knowledge could be used to develop novel preventive measures.

## Data Availability

Not applicable.

## Abbreviations

AGS: Aicardi-Goutières syndrome
AZT: azidothymidine (zidovudine)
CNV: copy number variation
ESC: embryonic stem cell
HERV: human endogenous retrovirus
HT: high-throughput
ICM: inner cell mass
iPSC: induced pluripotent stem cell
LINE-1: Long Interspersed Element-1
LTR: long terminal repeat
NRTI: nucleoside reverse transcriptase inhibitor
ORF: open reading frame
PGC: primordial germ cell
*RNP*: ribonucleoprotein particle
SINE: Short Interspersed Element
RT: reverse transcriptase
TPRT: target primed reverse transcription
